# An Evaluation of the Efficacy of Systemic Immune-Inflammation Index in Predicting Enterobius-Associated Appendicitis Preoperatively

**DOI:** 10.7759/cureus.36733

**Published:** 2023-03-27

**Authors:** Basak Erginel, Gokce Karli, Kadir Baziki, Neslihan Berker, Erbug Keskin, Feryal Gün Soysal

**Affiliations:** 1 Pediatric Surgery, Istanbul University School of Medicine, Istanbul, TUR; 2 Pathology, Istanbul University School of Medicine, Istanbul, TUR

**Keywords:** index, appendicitis, surgery, pediatric, enterobius vermicularis

## Abstract

Objective

Our study aimed to retrospectively evaluate *Enterobius*-associated appendicitis cases and compare them with acute appendicitis cases in terms of parameters such as the neutrophil-to-lymphocyte ratio (NLR), C-reactive protein (CRP)-to-lymphocyte ratio (CLR), platelet-to-lymphocyte ratio (PLR), and systemic immune-inflammation index (SII). We primarily aimed to evaluate the utility of SII in the differential diagnosis of *Enterobius*-associated appendicitis.

Methods

The appendectomy specimens of pediatric patients who were operated on for acute appendicitis between June 2016 and August 2022 were retrospectively evaluated. *Enterobius*-associated appendicitis cases were included for analysis. All patients were evaluated regarding age, gender, blood count, surgery, and pathology reports. Pathology reports were evaluated for the presence of histological signs of acute appendicitis. The patients were classified into an *Enterobius*-associated appendicitis group and a regular acute appendicitis group. CRP, white blood cell (WBC), red cell distribution width (RDW), neutrophils, lymphocytes, NLR, monocytes, eosinophils, platelet (PLT), PLR, CLR, and SII values were compared between the two groups.

Results

Eleven cases of *Enterobius*-associated appendicitis were identified out of 430 total cases (2.55%) examined. The mean age of the group with acute appendicitis was 12.83 ±3.16 years, while the mean age of the group with *Enterobius*-associated appendicitis was 8.55 ±2.54 years. There was no statistically significant difference in terms of CRP, WBC, RDW, lymphocytes, neutrophils, NLR, monocytes, eosinophils, PLT, PLR, and CLR values between the two groups (p>0.05). However, when the SII values of the participants were analyzed, it was observed that the SII values of the participants in the regular appendicitis group were significantly higher than those of the participants in the *Enterobius* group (p<0.05). Among the 11 *Enterobius*-associated appendicitis patients, seven appendectomy specimens revealed no inflammation and were regarded as negative appendectomy (63.63%).

Conclusion

This is the first study to demonstrate the utility of preoperative SII evaluation in *Enterobius*-associated appendicitis. SII is a simple, easy-to-calculate indicator of *Enterobius*-associated appendicitis and aids in the preoperative differential diagnosis of acute appendicitis.

## Introduction

*Enterobius vermicularis* *(E. vermicularis) *is one of the most prevalent infectious helminths in humans, with a higher incidence in children [1]. Its worldwide prevalence varies between 5 and 28% in children [2]. This parasite, also known as the pinworm, is transmitted through the fecal-oral route, and poor hand hygiene plays a significant role in the spread of the disease. After a person ingests the eggs, larvae emerge in the stomach or small intestine. The terminal ileum, ascending colon, particularly the cecum, and appendix are the main settlements of adult larvae. The female larvae migrate toward the anus at night and lay their eggs in that location. As a result, perianal itching, which is the most apparent clinical finding of the disease, increases at night. Other symptoms that may be seen are insomnia, irritability, chronic abdominal pain, and weight loss [3-5]. Scratching of the perianal area leads to recurrent auto-infection of the person via the infection’s presence on the hands of children with poor hand hygiene. *E. vermicularis* is a common parasite affecting more than 200 million people worldwide, especially children [6].

Acute appendicitis is the most common pathology requiring surgery in childhood [7]. The diagnosis is usually made clinically, and the patient is then prepared for surgery. Today, laparoscopic appendectomy is the preferred surgical method. Acute appendicitis due to *E. vermicularis *has been reported in some case reports and small case series [8,9]. The effect of the parasite on the formation of acute appendicitis is still a matter of controversy. Although inflammation secondary to the obliteration of the appendix vermiformis lumen by *E. vermicularis *itself or its eggs has been described, in some appendectomy specimens, no signs of inflammation were detected [10]. For this reason, it has been suggested that the presence of the parasite in the appendix may mimic an appendicitis-like picture [1]. Abdominal pain in the right lower quadrant and pelvis in the colic style, presumed to occur due to parasites in the lumen, has been defined as appendiceal colic, and it has been underlined that this perception might be responsible for many negative appendectomies [11,12]. Preoperative estimation of *E. vermicularis* is crucial, as the clinical picture caused by the parasite can be confused with acute appendicitis, thereby leading to unnecessary appendectomy. Thus, the spread of infection can be prevented by taking the necessary precautions during surgery [1]. Antibiotic prophylaxis is given for every acute appendicitis patient and, in the same way, anti-helminthic treatment can be immediately started for patients with *Entrobious-*associated appendicitis preoperatively. Its importance arises from the fact that it is one of the rare causes of appendicitis that needs medical treatment.

Although the correlation between acute appendicitis and parasitic infections and whether parasites are the cause of acute appendicitis is still debated, it is understood that many parasites and ova of parasites are encountered in the appendix lumen after appendectomy operations. Thus, the idea that parasitic infestations may be the underlying etiology of appendicitis should be kept in mind during differential diagnosis.

There are few case series and case reports of appendectomies with *E. vermicularis* infection in pediatric patients in the literature [13], and our series is one of the most extensive studies of acute appendicitis with *E. vermicularis* in pediatric patients. Unfortunately, no preoperative diagnostic marker for acute appendicitis with *E. vermicularis* infestation exists. In our study, by comparing ordinary acute appendicitis and appendicitis with *E. vermicularis* in terms of inflammatory biomarkers such as white blood cell (WBC) count, C-reactive protein (CRP), neutrophil-to-lymphocyte ratio (NLR), platelet-to-lymphocyte ratio (PLR), and the systemic immune-inflammation index (SII), we sought to predict the presence of *Enterobius* infestation in patients with acute appendicitis in an acute abdomen clinic.

SII (platelet x NLR) is a new marker of inflammation depending on three types of immune cells, and it is suggested to reflect the inflammation status [14,15]. To the best of our knowledge, there is no clinical research on the diagnostic value of SII in differentiating acute appendicitis patients from those with appendicitis with *E. vermicularis* in the pediatric population. SII might serve as a diagnostic biomarker in differentiating acute appendicitis from appendicitis with *E. vermicularis* in the pediatric population. This study aimed to review our experience in managing appendicitis with *E. vermicularis *and show the utility of SII in the differential diagnosis of acute appendicitis with *E. vermicularis.*

## Materials and methods

The appendectomy specimens of pediatric patients who underwent appendectomy for acute appendicitis between June 2016 and August 2022 were retrospectively evaluated. Eleven cases of *Enterobius*-associated appendicitis were identified out of 431 total cases (2.55%) examined. Patients under the age of 17 years who were operated on for acute appendicitis were included in the study. Preoperative hemogram and CRP tests of the patients were collected and NLR, PLR, and SII were calculated. SII is a marker of inflammation, which is calculated by combining platelet, neutrophil, and lymphocyte counts [14]. SII is calculated using the "platelet x neutrophil/lymphocyte" formula [15]. Patients with an accompanying chronic disease, those who used antibiotics before admission to the emergency department, and those who were found to have an incidental appendectomy during another operation were excluded from the study.

All patients were evaluated regarding age, gender, blood count, blood count ratios (CLR, NLR, SII), and surgery and pathology reports. The pathology reports were evaluated for the presence of histological signs of acute appendicitis. Acute transmural inflammation was considered necessary for the pathological diagnosis of acute appendicitis.

The patients were divided into the following two groups: the *Enterobius*-associated appendicitis group and the regular acute appendicitis group. CRP, WBC, RDW, neutrophils, lymphocytes, NLR, monocytes, eosinophils, platelets (PLT), PLR, CRP-to-lymphocyte ratio (CLR), and SII values were compared between the two groups.

Statistical analysis

Statistical analyses were performed using IBM SPSS Statistics version 22.0 (IBM Corp., Armonk, NY) for Windows. The Kolmogorov-Smirnov test was used to check the normal distribution of the results. Descriptive data were expressed as mean ± standard deviation (SD). For two-group comparisons, the Student's t-test was used for normally distributed data, and the Mann-Whitney U test was used for non-normally distributed data. A p-value <0.05 was considered statistically significant.

## Results

A total of 431 appendectomies were performed during the study period. *E. vermicularis* infestation was present in 11 appendicitis specimens (2.55%). The mean age of the patients in the acute appendicitis group was 12.10 ±1.70 years, while the mean age of the patients in the *Enterobius*-associated appendicitis group was 10.09 ±3.20 years. Of the 11 patients in the *Enterobius*-associated appendicitis group, six (54.54%) were males and five (45.45) were females. In the appendicitis group, 232 (55.23%) were males, and 188 (44.76) were females. The mean duration of abdominal pain was 2.18 ±0.69 days in the *Enterobius*-associated appendicitis group and 2.13 ±1.78 days in the acute appendicitis group. The mean length of hospital stay was 2.54 ±0.57 days in the *Enterobius*-associated appendicitis group and 2.53 ±1.84 days in the appendicitis group.

As shown in Table 1, there was no statistically significant difference in terms of age, gender, duration of abdominal pain (days), hospital stay (days), CRP, WBC, RDW, lymphocytes, neutrophils, NLR, monocytes, eosinophils, PLT, PLR, and CLR values ​​between the two groups (p>0.05). However, the SII values of the participants in the regular appendicitis group were found to be significantly higher than those of the participants in the *Enterobius* group (p<0.05).

**Table 1:** Comparison of demographic and laboratory characteristics between patients with acute appendicitis and those with Enterobius-associated appendicitis *P-value significant at the 0.05 level CRP: C-reactive protein; WBC: white blood cell; NLR: neutrophil-to-lymphocyte ratio; PLT: platelets; PLR: platelet-to-lymphocyte ratio; CLR: C-reactive protein-to-lymphocyte ratio; SII: systemic immune-inflammation index (platelet x NLR); SD: standard deviation

Variables		*Enterobius* (n=11)	Acute appendicitis (n=420)	P-value
Age, years, mean ±SD		12.10 ±1.70	10.09 ±3.20	0.103
Gender, n (%)	Female	5 (45.45)	188 (44.76)	0.972
	Male	6 (54.54)	232 (55.23)	
Duration of abdominal pain, days, mean ±SD		2.18 ±0.69	2.13 ±1.78	0.243
Length of hospital stay, days, mean ±SD		2.54 ±0.57	2.53 ±1.84	0.145
CRP, mean ±SD		52.32 ±65.83	14.56 ±10.28	0.440
WBC, mean ±SD		14712.76 ±6318.95	13503.64 ±5552.88	0.581
RDW, mean ±SD		13.79 ±1.20	14.62 ±1.82	0.087
Lymphocytes, mean ±SD		3083.64 ±1950.54	1791.38 ±884.52	0.067
Neutrophils, mean ±SD		10487.27 ±4759.19	11960.45 ±5862.77	0.462
NLR, mean ±SD		5.75 ±6.05	7.97 ±5.68	0.051
Monocytes, mean ±SD		912.73 ±295.33	832.76 ±491.94	0.255
Eosinophils, mean ±SD		254.27 ±242.03	72.07 ±92.05	0.052
PLT, mean ±SD		313181.82 ±65862.07	296906.90 ±96791.50	0.611
PLR, mean ±SD		184.19 ±108.46	162.08 ±105.50	0.308
CLR, mean ±SD		0.01 ±0.00	0.04 ±0.05	0.256
SII (platelet x NLR), mean ±SD		1369.27 ±1488.16	2441.93 ±1861.67	0.038*

Among the 11 *Enterobius*-associated appendicitis patients, seven appendectomy specimens revealed no inflammation and were regarded as negative appendectomy (63.63%). Among 420 patients operated on for acute appendicitis, nine patients' histological examinations revealed no inflammation and were regarded as negative appendectomy (2.14%). Table 2 presents the histological analysis and existence of inflammation.

**Table 2:** Histological analysis of appendices

	*Enterobius*-associated appendicitis (n=11)	Acute appendicitis (n=420)
Inflammation	4	411
No inflammation	7	9

## Discussion

Acute appendicitis is one of the most common pediatric emergency surgical pathologies. Today, complete healing can be achieved with a simple surgical intervention that can also be performed laparoscopically. The pathophysiology of acute appendicitis includes the obstruction of the lumen of the appendix. This obstruction can be due to fecolith, lymphoid hypertrophy, fruit seeds, or parasites [16]. *E. vermicularis* is the most common parasite in appendicitis specimens. However, no biomarker is yet available to predict *Enterobius*-associated appendicitis preoperatively. Recently described laboratory serum biomarkers ratios, such as NLR, CLR, PLR, and SII, have provided promising results in the prediction of immune-inflammatory situations [17]. In this study, we sought to investigate the clinical values of these indexes, especially the SII, a recently defined biomarker based on peripheral lymphocyte, neutrophil, and PLT counts. Our study aimed to retrospectively evaluate *Enterobius*-associated appendicitis by conducting one of the most extensive series in children, comparing these cases with routine acute appendicitis in terms of blood values, as well as evaluating the utility of various preoperative indexes such as the SII. It is essential to develop an index that can be calculated using simple routine preoperative blood tests to enable us to predict whether parasites are involved in the etiology of appendicitis, as some studies have shown the abdominal cavity being contaminated with *Enterobius* after appendectomy [18].

Debates are still ongoing about the relationship between *Enterobius* and acute appendicitis [19]. However, as mentioned in the study by Sousa et al., there are many similarities in the clinical findings between *Enterobius*-associated appendicitis and simple acute appendicitis, and it is challenging to differentiate between them preoperatively [19]. They have found an incidence of *Enterobius*-associated appendicitis of 1.07%, while we identified a rate of 2.55%, and this could be attributed to the fact that our study was conducted in a developing country.

In many studies, no inflammation was observed in the appendectomy specimens of *Enterobius*-associated appendicitis [20]. This could indicate that the parasite enters the appendix lumen and causes clinical findings similar to appendicitis but does not always cause transmural inflammation in the lumen as in acute appendicitis [20]. Therefore, in terms of evaluating the presence of inflammation, an inflammatory index such as SII may be highly useful in preoperative diagnosis.

The absence of inflammation in appendix specimens in *Enterobius*-associated appendicitis means that the negative appendectomy rate is high in patients with *E. vermicularis* infestation. In one of the most extensive series ever published, Fleming et al. found that the rate of negative appendectomy in patients with *Enterobius*-associated appendectomy was 69.2% [21]. Our study found no inflammation in the appendix specimens in eight of our 11 patients with *Enterobius*-associated appendicitis. In other words, inflammation was not observed in 63.63% of our patients with *Enterobius*-associated appendicitis and had a negative appendectomy performed. This is a relatively high rate, highlighting the value of SII in the differential diagnosis.

We found no significant difference between the group with *Enterobius*-associated appendicitis and that with regular acute appendicitis regarding blood count parameters except SII. Although the CRP level was higher in *Enterebious*-associated appendicitis than in acute appendicitis in most of the case reports in the literature, we found no significant difference between our two groups [22] Although there were differences between mean CRP levels of *Enterobius*-associated and regular acute appendicitis groups (CRP levels were higher in the *Enterobius* group), it was observed that the SD values ​​were high. This shows that the mean values ​​account for a wide range of values. Therefore, although there was a difference between the means, the p-value was above 0.050. Analysis results have been checked in SPSS output files and it is seen that the values ​​are correct. Therefore, the difference in CRP between the groups was not statistically significant. The same scenario was observed in terms of the eosinophil count of the two groups, as there was no statistically significant difference between the groups. However, Akkapulu et al. have reported a significant difference in terms of WBC between two such groups in their study [23]. According to their study, the WBC count was significantly higher among the group with acute appendicitis. The difference could be attributed to the fact that while Akkapulu et al. studied adult patients, our study involved pediatric patients.

## Conclusions

To the best of our knowledge, this was one of the first studies to demonstrate the utility of preoperative SII evaluation in *Enterobius*-associated appendicitis. *Enterobius*-associated appendicitis should be kept in the differential diagnosis of acute appendicitis. Early diagnosis of this condition will help reduce the number of negative appendectomies. This index can be routinely studied in the preoperative period in pediatric acute appendicitis patients. SII is a simple, non-invasive, easy-to-calculate indicator of *Enterobius*-associated appendicitis and aids in the preoperative differential diagnosis of acute appendicitis.
